# Editorial: Calcium and pulmonary hypertension

**DOI:** 10.3389/fphys.2022.1019158

**Published:** 2022-11-03

**Authors:** Ji-Feng Li, Yu-Qin Chen, Lan Wang, Yun-Shan Cao, Jason X.-J. Yuan

**Affiliations:** ^1^ Department of Respiratory and Critical Care Medicine, Beijing Chao-Yang Hospital, Capital Medical University, Beijing, China; ^2^ State Key Laboratory of Respiratory Disease, National Clinical Research Center for Respiratory Disease, The First Affiliated Hospital of Guangzhou Medical University, Guangzhou, Guangdong, China; ^3^ Department of Respiratory and Critical Care Medicine, West China Hospital, Sichuan University, Chengdu, China; ^4^ Department of Cardiology, Gansu Provincial Hospital, Lanzhou, China; ^5^ Division of Pulmonary, Critical Care and Sleep Medicine, Department of Medicine, University of California, San Diego, San Diego, CA, United States

**Keywords:** calcium channel blockers (CCBs), calcium, pulmonary vasoreactivity testing, pumonary hypertension, gene

Calcium channel blockers (CCBs) have been used in the treatment of pulmonary hypertension since 1980s (Rubin, 1985). In 1992, a study showed that high doses of CCBs in patients with primary pulmonary hypertension who respond with reductions in pulmonary-artery pressure (PAP) and pulmonary vascular resistance might improve survival over a five-year period (Rich et al., 1992). Subsequently, a number of studies explored the impact of CCBs to the outcome of pulmonary hypertension, especially pulmonary arterial hypertension (PAH). At present, many guidelines suggested that pulmonary vasoreactivity testing should be done in some kinds of pulmonary arterial hypertension and responders are suggested to be treated with high dose CCBs (Galie et al., 2015).

A positive acute response is defined as a reduction of the mean PAP ≧10 mmHg to reach an absolute value of mean PAP ≦40 mmHg with an increased or unchanged cardiac output (Ruopp and Cockrill, 2022). Studies showed that long-term CCBs responders displayed a more pronounced fall in mean PAP and reached an absolute value of mean PAP lower than that measured in non-responders (Sitbon et al., 2005). Whole-exome sequencing showed that gene variants differed in vasodilator-responsive idiopathic PAH *versus* vasodilator-nonresponsive idiopathic PAH (Hemnes et al., 2016). More biomarkers should be explored to detect vasodilator-responders early and easily, including in other types of pulmonary hypertension (He et al.). The recent research of this Topic showed that TIMP-1 elevation could serve as a circulating biomarker to identify PH among COPD patients.

The recent work highlighted multiple ways to predict the long-term outcome of pulmonary hypertension patients. This Research Topic includes a study which found that besides hemodynamics, exercise capacity was valuable in the evaluation of the long-term outcome of pulmonary hypertension patients (Jiang et al.). This recent research showed that exercise-based rehabilitation (aerobic exercise training) may benefit the change from baseline at Week 26 in right ventricular stoke volume (RVSV), determined by pulmonary artery from CMR.

Besides the CCBs that have been predominantly used like diltizem, nifedipine and amlodipine, other medicine related to calcium signaling have also been used in pulmonary hypertension (Humbert et al., 2022). Levosimendan, a calcium sensitizing agent with positive inotropic and vasodilatory effects, holds promise for patients with pulmonary hypertension and right heart failure (Hoeper and Granton, 2011; Coz Yataco et al., 2016) (Qu et al.). This recent research showed that Levosimendan treatment could effectively improve acute decompensated right heart failure and systemic hemodynamics in connective tissue disease-associated pulmonary arterial hypertension patients, with positive effects on survival in hospital.

Many gene mutations are involved in the etiology of pulmonary hypertension such as BMPR2. In this topic, we reported a rare case of pulmonary hypertension caused by Kartagener’s syndrome with a novel homozygous nonsense mutation in CCDC40 gene (Dai et al.). This finding expanded the mutational spectra of CCDC40 gene related Kartagener’s syndrome which is vital for screening and genetic diagnosis of the disease.

Calcium is involved in the etiology, mechanism and therapy target in pulmonary hypertension. CCBs can be used in first diagnosed pulmonary arterial hypertension and calcium sensitizing agent can be used in the late-stage pulmonary hypertension with right heart failure. More researches should be investigated in the future, such as: whether CCBs can be used in other types of pulmonary hypertension? are there calcium signaling related gene mutation or biomarkers are related to pulmonary hypertension? whether new drugs can be developed targeting calcium signaling such as calcium channels or calcium related receptors?
